# Contrast-enhanced US Bosniak Classification: intra- and inter-rater agreement, confounding features, and diagnostic performance

**DOI:** 10.1186/s13244-024-01858-7

**Published:** 2024-11-29

**Authors:** Dong-dong Jin, Bo-wen Zhuang, Ke Lin, Nan Zhang, Bin Qiao, Xiao-yan Xie, Xiao-hua Xie, Yan Wang

**Affiliations:** Department of Medical Ultrasonics, Institute of Diagnostic and Interventional Ultrasound, The First Affiliated Hospital of Sun Yat-Sen University, No.58 Zhongshan Road 2, Guangzhou, 510080 People’s Republic of China

**Keywords:** Bosniak classification, Cystic renal masses, Contrast-enhanced US, Reproducibility, Diagnostic performance

## Abstract

**Background:**

The contrast-enhanced US (CEUS) Bosniak classification, proposed by the European Federation for Ultrasound in Medicine and Biology (EFSUMB) in 2020, predicts malignancy in cystic renal masses (CRMs). However, intra- and inter-rater reproducibility for CEUS features has not been well investigated.

**Purpose:**

To explore intra- and inter-rater agreement for US features, identify confounding features, and assess the diagnostic performance of CEUS Bosniak classification.

**Materials and methods:**

This retrospective study included patients with complex CRMs who underwent CEUS examination from January 2013 to August 2023. Radiologists (3 experts and 3 novices) evaluated calcification, echogenic content, wall, septa, and internal nodules of CRMs using CEUS Bosniak classification. Intra- and inter-rater agreements were assessed using the Gwet agreement coefficient (Gwet’s AC). Linear regression identified features associated with discrepancies in Bosniak category assignment. Diagnostic performance was evaluated using the area under the receiver operating characteristic curve (AUC).

**Results:**

A total of 103 complex CRMs were analyzed in 103 patients (mean age, 50 ± 15 years; 66 males). Intra-rater agreement for the Bosniak category was substantial to almost perfect (Gwet’s AC 0.73–0.87). Inter-rater agreement was substantial for the Bosniak category (Gwet’s AC 0.75) and moderate to almost perfect for US features (Gwet’s AC 0.44–0.94). Nodule variation (i.e., absence vs. obtuse margin vs. acute margin) explained 84% of the variability in the Bosniak category assignment. CEUS Bosniak classification showed good diagnostic performance, with AUCs ranging from 0.78 to 0.90 for each rater.

**Conclusions:**

CEUS Bosniak classification demonstrated substantial intra- and inter-rater reproducibility and good diagnostic performance in predicting the malignancy potential of CRMs. Nodule variations significantly predicted differences in Bosniak category assignments.

**Critical relevance statement:**

Contrast-enhanced US Bosniak classification reliably predicts malignancy in cystic renal masses, demonstrating substantial reproducibility and diagnostic accuracy. This improves clinical decision-making and patient management.

**Key Points:**

- Intra- and inter-rater reproducibility for contrast-enhance US features for Bosniak classification have not been well investigated.
- Substantial inter-rater agreements for the Bosniak category and variable agreements for determining imaging features were found.
- Contrast-enhanced US Bosniak classification is reproducible and has good diagnostic performance for predicting malignancy in cystic renal masses.

**Graphical Abstract:**

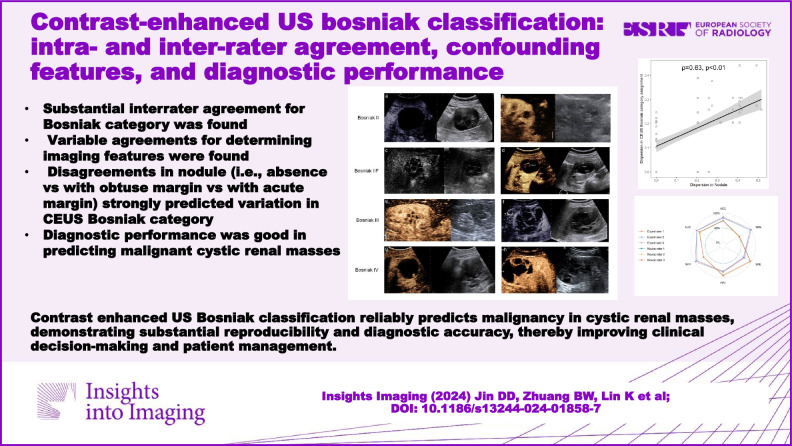

## Introduction

Cystic renal masses (CRMs) are highly prevalent, with an estimated occurrence of 55% in individuals over 70 years old [1]. When a cyst has any of the following characteristics: septations within the cyst, calcifications, thickening of the walls, enhancement, and/or soft tissue components, it is considered a complex CRM [2–4]. Unlike the vast majority of simple CRMs that are benign [5], complex cysts, which account for 8–15% of the CRMs, have a pooled malignancy rate of 24% [6, 7]. Due to varying levels of complexity, these masses present distinct malignancy risks and require different management strategies, making the estimation of malignant potential in complex CRMs crucial [3].

To stratify the risk of malignancy in complex CRMs, the Bosniak classification system was developed and has since been widely adopted in clinical practice [2, 8, 9]. Although Bosniak classification was initially proposed for CT, the classification criteria have been successfully utilized in both research and clinical settings for contrast-enhanced US (CEUS) [10–17]. According to previous studies [12, 18, 19], CEUS is more likely to provide a higher classification for CRMs than CT/MRI when applying the same Bosniak criteria, owing to its ability to visualize very thin septa and small protrusions. Therefore, in an attempt to enhance the ability of CEUS to more accurately evaluate CRMs, experts from the European Federation of Societies for Ultrasound in Medicine and Biology (EFSUMB) have adapted the Bosniak classification system for use in CEUS. This adaptation is known as the CEUS Bosniak classification [4]. In this adapted classification, CRMs are categorized into five categories (I, II, IIF, III, and IV) based on ultrasound features, including echogenic content, calcifications, enhancement of septa, wall thickness, and protrusions. A few previous studies [14, 15, 20] have shown that the CEUS Bosniak classification exhibited moderate inter-rater agreement, moderate to almost perfect intra-rater agreement, and good correlation with pathological results. However, such studies focused solely on the Bosniak category, neglecting the detailed imaging features. Moreover, it has not been previously investigated which US features are associated with inter-rater variability in the CEUS Bosniak classification.

Therefore, the main purpose of our study was to investigate the classification agreement of each detailed feature associated with CEUS Bosniak category assignment and identify features associated with inter-rater disagreement in CEUS Bosniak category assignment. Additionally, we explore the diagnostic performance of CEUS Bosniak classification in identifying malignancies within the complex CRMs.

## Materials and methods

This single-center and retrospective study was approved by our institutional review board. Written informed consent was waived due to the retrospective nature. Six radiologists followed the Guidelines for Reporting Reliability and Agreement Studies when assessing CRMs [21]. The Strengthening the Reporting of Observational Studies in Epidemiology guidelines is applicable to our study [22].

### Study design

To assess the reproducibility of image interpretation in the classification of complex CRMs, six radiologists (three with over 5 years of abdominal ultrasound (US) experience and three with < 2 years of abdominal US experience were recruited as raters. To assess the reproducibility of CEUS Bosniak classification, the results of image analysis from all raters were compared to determine intra- and inter-rater agreement on each detailed feature and Bosniak category. In order to guide further modifications of Bosniak classification for improved inter-rater agreement, feature scoring, and Bosniak category assignment were investigated to identify features that could predict the inter-rater disagreement. Furthermore, we explored the diagnostic performance of the CEUS Bosniak category in detecting malignant complex CRMs among different raters.

### Patients

**Fig. 1:**
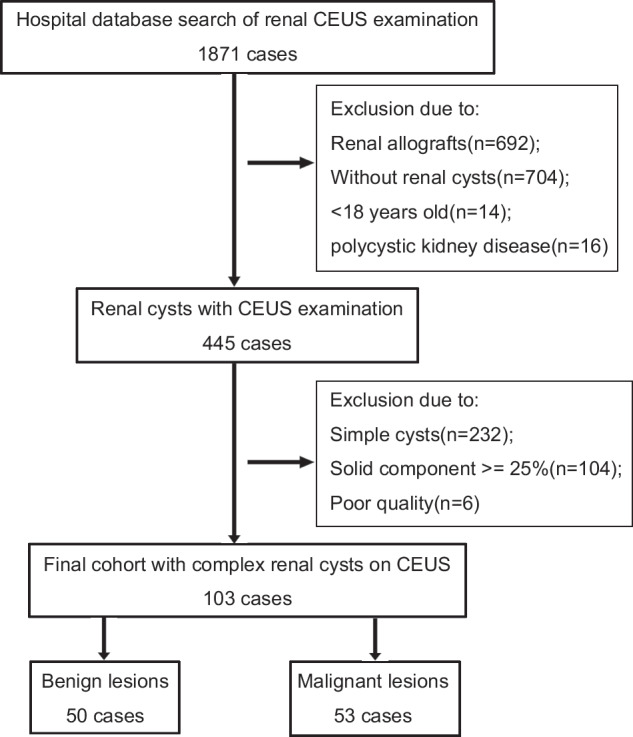
Flowchart of patient inclusion. CEUS, contrast-enhanced US

The study sample was composed of patients who were diagnosed with complex CRMs based on US and cross-sectional imaging (CT or MRI) from January 2013 to August 2023. Inclusion criteria were: (a) all patients underwent CEUS examination and cross-sectional imaging with an interval of no more than 6 months; (b) CRMs were diagnosed in accordance with Bosniak classification for CT/MRI [2] and CEUS [4]; (c) CRM with pathology reports within 6 months after CEUS examination, or with at least 2-year follow-up; (d) age over 18 years old. Exclusion criteria were: (a) simple renal cyst without any septa, thickened wall, calcification, echogenic content, or internal solid enhancement; (b) images with poor quality and severe visualization constraints; (c) polycystic kidney disease; (d) enhancing tissue > 25% of the renal lesion. CRM with more than 25% of enhancing tissue was excluded because it was probably an aggressive solid renal mass with necrosis and not deemed a cystic lesion [2, 23]. The flowchart of included patients is presented in Fig. 1.

### US and CEUS protocols

All CRMs were scanned initially on B-mode US (BUS). For patients with more than one CRM, we selected the most complex mass as the target lesion. After localizing target observations, contrast agents (SonoVue) were administered. Images were obtained and stored for later analysis. Further details of our imaging protocol are provided in Appendix E1.

### Interpretation of BUS and CEUS images

According to CEUS Bosniak classification, the detailed features associated with the Bosniak category were (1) echogenic content: presence or absence of internal echogenic content or debris which is visible on BUS but not seen on CEUS; (2) calcification: presence or absence of calcification of the septa or wall, and whether calcification could hamper evaluation of CRM; (3) septation: the number, thickness, and regularity of enhancing septa; (4) wall: the thickness and regularity of enhancing wall; (5) nodule: presence or absence of internal solid enhancement, as well as its size and margin (with obtuse margin or with acute margin).

Following the CEUS Bosniak classification, we defined the BUS Bosniak classification, which included features such as echogenic content, calcification, septation, wall, and nodule, regardless of whether these features showed enhancement. Unless otherwise specified, the features mentioned below are those outlined in the CEUS Bosniak classification.

**Fig. 2:**
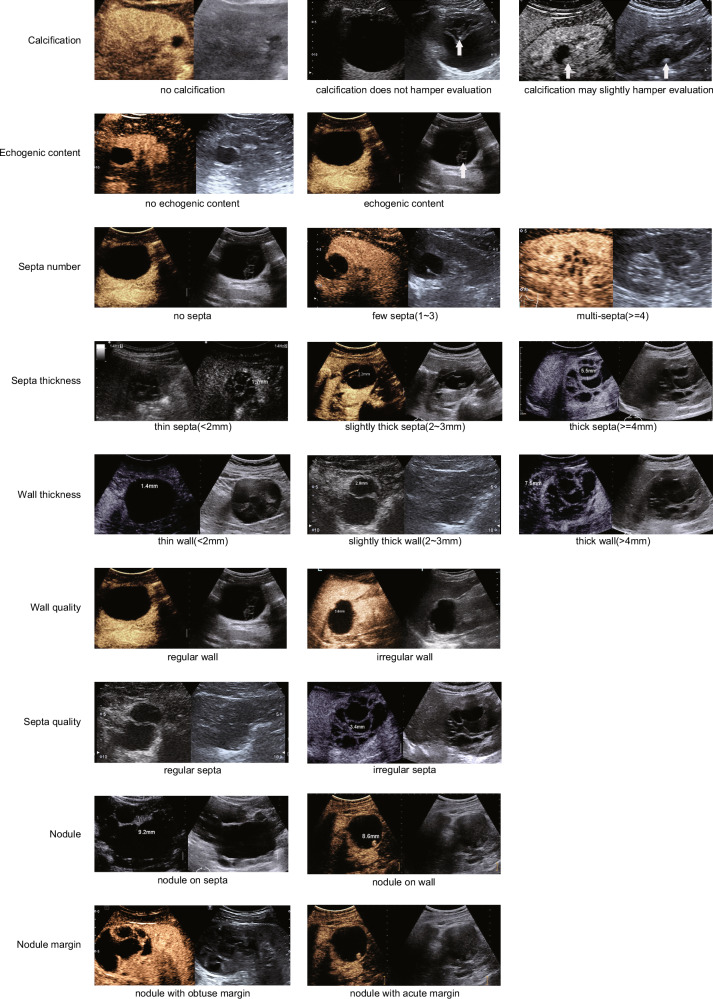
Contrast-enhanced US (CEUS) images demonstrate the determining features mentioned in the CEUS Bosniak classification. The left side of each image is contrasted, and the right side is non-contrasted

The evaluation of images took place in two sessions. The first session recorded the following features: (1) BUS: echogenic content, calcification, septation, wall, and internal solid tissue; (2) CEUS: enhancement of septa, wall, and nodule. The second session recorded: (1) BUS: echogenic content, calcification; (2) CEUS: enhancement of septa, wall, and nodule. To minimize recall bias, a 1-month interval was set between the two evaluations, and the patient order was randomized. Before the evaluation, all raters were provided with CEUS Bosniak classification (Fig S1.a), the mind map for classification rating (Fig S1.b), and a 1-h training with several typical image examples (Fig. 2). Six raters independently assessed all imaging features and assigned Bosniak category to complex CRMs while blinded to histopathologic findings and the assessments of other raters.

### Diagnostic reference standard

The diagnosis of CRMs, either malignant or benign, is based on pathologic confirmation (biopsy or resection), as well as evaluations of morphology, mass size, and nodule volume. Specifically, any change in these characteristics confirms the diagnosis of malignant CRMs, whereas their stability over at least two years confirms the diagnosis of benign CRMs [13].

### Statistical analysis

The distributions of scoring for imaging features and Bosniak category assignment are presented by raters as numbers. Gwet’s agreement coefficient (Gwet’s AC) was calculated to assess the intra- and inter-rater agreement among all raters as it is an excellent choice for an unbalanced dataset and demonstrates reduced susceptibility to prevalence-based kappa paradoxes, such as situations where there are poor Kappa statistics but high agreement [24, 25]. The degree of agreement was interpreted according to the scale developed by Landis and Koch (coefficient value: < 0, poor agreement; 0–0.20, slight agreement; 0.21–0.40, fair agreement; 0.41–0.60, moderate agreement; 0.61–0.80, substantial agreement; and 0.81–1.00, almost perfect agreement) [26].

Dispersion indices were computed for each feature as the ratio of the rater assignment standard deviation to the potential value range. Univariable and multivariable linear regression was employed subsequently to formulate a model showing how the dispersion of detailed imaging features affects the variability in Bosniak category assignments. After identifying features that may adversely impact reliability, potential improvements can be made to enhance the clinical applicability of CEUS Bosniak classification system. Spearman rank correlation (*ρ*) was calculated to quantify the effects of each feature on Bosniak classification. Post hoc power analysis was conducted by using the fixed sample size (*n* = 103) and the fixed effect size (*f* ^2^ = 0.15) for our linear model.

The diagnostic performance of each rater for malignant CRMs was evaluated using the area under the receiver operating characteristic curve (AUC), as well as the accuracy, sensitivity, specificity, positive predictive value (PPV), and negative predictive value (NPV). The diagnostic performance was compared between different raters and between BUS and CEUS Bosniak classification using the DeLong test.

*p*-value < 0.05 is considered statistically significant. Analyses were conducted using R software (version 4.3.2) and SPSS (version 26).

## Results

### Patient demographics

Out of 1871 patients with renal CEUS, 103 patients (mean age, 50 years ± 15 years (SD); 66 were male) with 103 masses were included in the study (Fig. 1). Of the 103 complex CRMs, 51.5% (53 of 103) were malignant and 48.5% (50 of 103) were benign. Malignancies primarily consist of clear cell renal cell carcinomas (ccRCCs) (*n* = 41/53, 77.4%) and papillary renal cell carcinomas (pRCCs) (*n* = 8/53, 15.1%). More details are presented in Table 1.

**Table 1:** Clinical and pathologic characteristics of patients

Clinical feature	Total (*n* = 103)	Benign group (*n* = 50)	Maligant group (*n* = 53)	*p-*value
Age (mean ± SD)	50.1 ± 14.9	48.1 ± 13.8	51.9 ± 15.9	0.20
Sex				0.02
Male	66	26	40	
Female	37	24	13	
Cyst size (cm, mean)	3.4	3.0	3.9	
Site				0.55
Upper pole	38	19	19	
Middle	17	10	7	
Lower	48	21	27	
Side				1
Left	49	24	25	
Right	54	26	28	
Diagnostic confirmation				< 0.001
Radical nephrectomy	39	8	31	
Partial nephrectomy	32	11	21	
Cyst decapitating decompression operation	7	7	0	
Ablation	7	6	1	
Clinical follow-up	18	18	0	
Final diagnosis^a^				
Benign lesion	50	50	…	
ccRCC	41	…	36	
pRCC	8	…	8	
Other malignancy	4	…	9	

Benign lesions included benign simple cysts (*n* = 20), follow-up (*n* = 21), cystic nephroma (*n* = 4), and epithelioid lipid-poor angiomyolipoma (*n* = 5); Other malignancies included tubulocystic renal cell carcinoma (*n* = 1), multilocular cystic renal cell carcinoma (2) and clear cell sarcoma (*n* = 1)

*SD* standard deviation, *ccRCC* clear cell renal cell carcinoma, *pRCC* papillary renal cell carcinoma

^a^ Pathology results were obtained from pathologic confirmation or clinical follow-up

### Evaluation of US features

After the two sessions of evaluation, 1236 assessments by 6 readers (3 expert raters, 3 novice raters) were completed for scoring all complex CRMs. Distribution of scoring by raters for detailed features is shown in Table S1 and Table S2.

### Intra-rater agreement

Table S3 illustrates the intra-rater agreement of each feature in complex CRMs across two rounds of evaluation by 6 raters. There was substantial to almost perfect agreement for the Bosniak category (Gwet’s AC range, 0.73–0.87) and moderate to almost perfect agreement for detailed imaging features (Gwet’s AC range, 0.60–0.91).

### Inter-rater agreement

In regard to the Bosniak category assignment (Table 2), the inter-rater agreement was substantial among all raters (Gwet agreement coefficient, 0.75; 95%CI, 0.70 to 0.80), with the agreement higher for expert raters than for novice raters (Gwet’s AC, 0.85 vs. 0.77, respectively, *p* = 0.04). In regard to US features, the inter-rater agreement ranged from moderate to almost perfect (Gwet’s AC range, 0.44–0.94). Expert raters showed better agreement than novice raters in the assessment of septa thickness (Gwet’s AC, 0.82 vs. 0.73), wall thickness (Gwet’s AC, 0.60 vs. 0.40), and septa /wall quality (Gwet’s AC, 0.74 vs. 0.54) (*p* < 0.05 for all). For both expert and novice raters, imaging feature agreement was lowest for wall thickness (Gwet’s AC: 0.60 for experts, 0.40 for novices) and highest for echogenic content (Gwet’s AC: 0.95 for experts, 0.91 for novices).

**Table 2:** Interobserver agreement of US features

Feature	Interobserver agreement			*p*
	All raters (*n* = 6)	Expert raters (*n* = 3)	Novice raters (*n* = 3)	Expert raters vs. Novice raters
Bosniak category	0.75 (0.70, 0.80)	0.85 (0.80, 0.91)	0.77 (0.72, 0.83)	0.04
BUS				
Calcification	0.86 (0.81, 0.92)	0.87 (0.81, 0.93)	0.84 (0.76, 0.91)	0.46
Echogenic content	0.94 (0.90, 0.98)	0.95 (0.91, 0.99)	0.91 (0.86, 0.97)	0.31
CEUS				
Septa number	0.83 (0.79, 0.87)	0.86 (0.81, 0.91)	0.79 (0.74, 0.85)	0.09
Septa thickness	0.76 (0.72, 0.80)	0.82 (0.77, 0.87)	0.73 (0.67, 0.78)	0.01
Wall thickness	0.50 (0.40, 0.59)	0.60 (0.49, 0.72)	0.40 (0.27, 0.53)	0.02
Presence of nodule	0.67 (0.58, 0.77)	0.81 (0.72, 0.90)	0.70 (0.59, 0.82)	0.15
Nodular margin: obtuse /acute	0.79 (0.72, 0.87)	0.84 (0.77, 0.92)	0.84 (0.77, 0.91)	0.92
Septa or wall quality	0.44 (0.34, 0.55)	0.74 (0.63, 0.84)	0.54 (0.41, 0.67)	0.02

Data are Gwet agreement coefficient; numbers in parentheses are 95% confidence intervals

*p*-values were obtained from two-tailed independent sample *z*-tests (based on asymptotic normality)

*US* ultrasound, *BUS* B-mode ultrasound, *CEUS* contrast-enhanced ultrasound

**Fig. 3:**
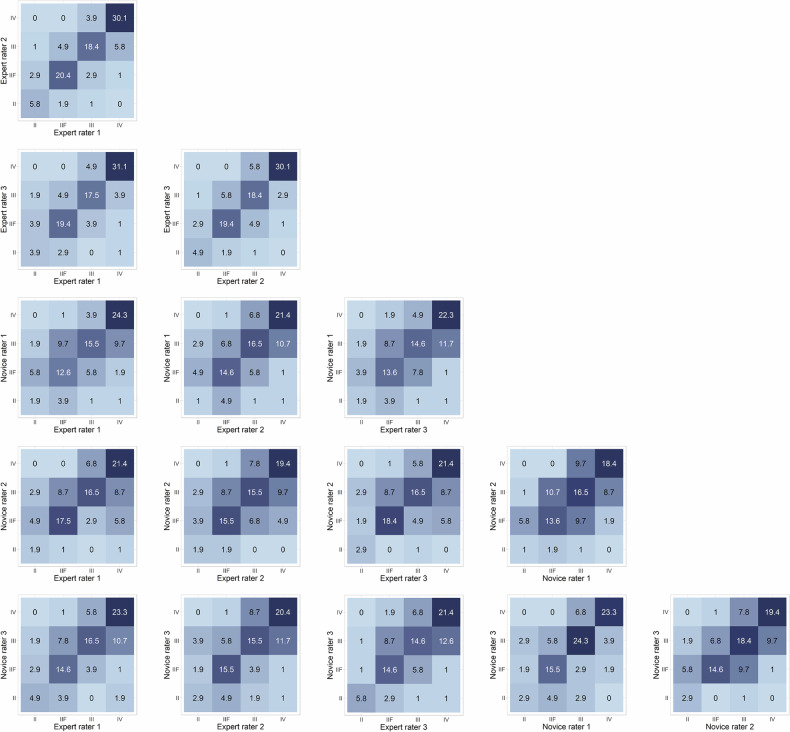
Confusion matrices of rater-rater pairings for the Bosniak category. The horizontal and vertical axes represent the Bosniak category given by the raters. Numbers represent percentages

Confusion matrices illustrate the correlation between any two raters for the Bosniak category assignment (Fig. 3 and Table S4). A noticeable trend shows that the consistency between any two raters increases with a higher Bosniak score. The inter-rater agreement for US features between any two raters is shown in Tables S5 to S12.

### Confounding features

Confounding features refer to those features that negatively impact the inter-rater agreement on CEUS Bosniak classification. Univariable and multivariable linear regression analyses were performed to predict the dispersion in Bosniak category assignment by imaging feature disagreement, and the result is shown in Table 3. Nodule (i.e., absence vs. with obtuse margin vs. with acute margin) accounted for the greatest variability in both unadjusted models (*p* < 0.001, 54% explained) and adjusted model (*p* < 0.001, 84% total variability explained). The power of the test was 98.8%, indicating that our sample size was sufficient to identify confounding features.

**Table 3:** Linear regression models of dispersion in Bosniak category predicted by dispersion in associated features

Model	Unadjusted			Adjusted			
	*β* coefficient (95% CI)	*p-*value	Partial *η*^2^	*β* coefficient (95% CI)	*p*-value	Type III partial *η*^2^	partial *η*^2^
CEUS Bosniak classification (*n* = 103)							0.84
Septa number	0.08 (− 0.11, 0.27)	0.43	0.03	0.02 (− 0.14, 0.19)	0.80	0.16	
Septa thickness	− 0.06 (− 0.35, 0.23)	0.7	0.11	− 0.13 (− 0.37, 0.12)	0.31	0.30	
Wall thickness	0.01 (− 0.17, 0.18)	0.95	0.15	0.00 (− 0.14, 0.14)	0.99	0.37	
Calcification	0.02 (− 0.10, 0.13)	0.78	0.06	− 0.03 (− 0.13, 0.07)	0.55	0.33	
Echogenic content	0.09 (− 0.07, 0.24)	0.27	0.01	0.13 (− 0.00, 0.27)	0.06	0.24	
Septa or wall quality	0.04 (− 0.06, 0.13)	0.44	0.01	− 0.01 (− 0.09, 0.07)	0.73	0.18	
Nodule	0.38 (0.27, 0.48)	< 0.001	0.54	0.39 (0.29, 0.50)	< 0.001	0.73	

*CEUS* contrast-enhanced ultrasound

**Fig. 4:**
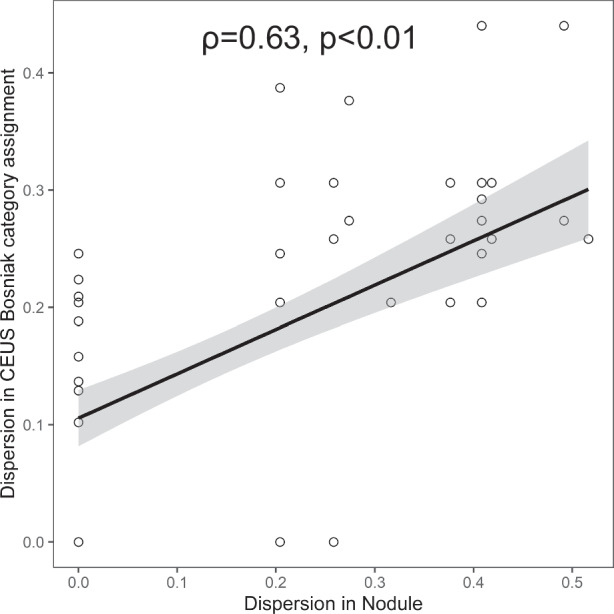
Linear regression shows dispersion in Bosniak category assignment as a function of dispersion in nodule (i.e., absence vs. with obtuse margin vs. with acute margin) on contrast-enhanced US (84% total variability explained; *ρ* = 0.63, *p* < 0.01)

Figure 4 demonstrates univariable linear regression in Bosniak category assignment as dependent on dispersion in nodules (54% explained). It shows that variance in nodule was a strong predictor of variance in Bosniak category assignment. The linear regressions for other features are presented in Supplementary Fig. S2, suggesting that these features cannot serve as the predictor of inter-rater variability (all *p* > 0.05).

### Diagnostic performance of CEUS Bosniak classification

**Fig. 5:**
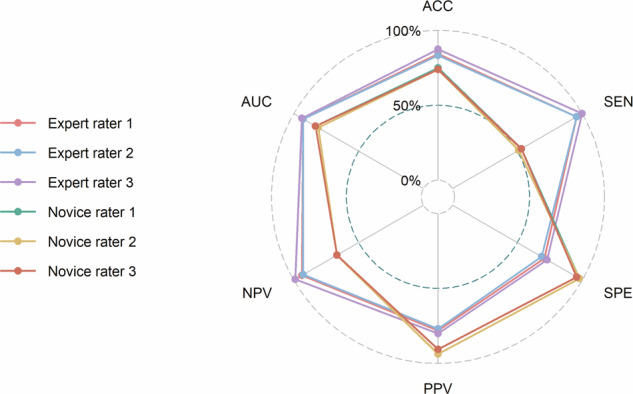
Radar chart shows the diagnostic performance of the Bosniak category by each rater

CEUS Bosniak classification showed good diagnostic efficiency with AUC ranging from 0.78 to 0.90 across the six raters (Fig. 5 and Table 4). The AUCs of experts were superior to those of novices (AUC: 0.89, 0.89, 0.90 for experts vs. 0.78, 0.80, 0.80 for novices; *p* < 0.05 in all expert-novice pairings) (Table 4 and Table S13). The diagnostic performance of each feature by each rater is presented in Supplementary Table S14 and Fig. S3.

**Table 4:** Diagnostic performance in the detection of malignancy in CRMs

Rater	AUC	Accuracy (%)	Sensitivity (%)	Specificity (%)	PPV (%)	NPV (%)
All CRMs						
Expert raters						
Rater 1	0.89 [0.83, 0.95]	81 [80, 81] (83/103)	92 [85, 100] (49/53)	68 [55, 81] (34/50)	75 [65, 86] (49/65)	90 [80, 99] (34/38)
Rater 2	0.89 [0.83, 0.94]	80 [79, 80] (82/103)	92 [85, 100] (49/53)	66 [53, 79] (33/50)	74 [64, 85] (49/66)	89 [79, 99] (33/37)
Rater 3	0.90 [0.84, 0.96]	84 [83, 84] (86/103)	96 [91, 100] (51/53)	70 [57, 83] (35/50)	77 [67, 87] (51/66)	95 [87, 102] (35/37)
Novice raters						
Rater 4	0.80 [0.72, 0.88]	72 [72, 72] (74/103)	51 [38, 64] (27/53)	94 [87, 100] (47/50)	90 [79, 101] (27/30)	64 [53, 75] (47/73)
Rater 5	0.78 [0.70, 0.86]	71 [70, 71] (73/103)	49 [36, 62] (26/53)	94 [87, 100] (47/50)	90 [79, 101] (26/29)	64 [52, 74] (47/74)
Rater 6	0.80 [0.71, 0.88]	71 [70, 71] (73/103)	51 [38, 64] (27/53)	92 [84, 100] (46/50)	87 [75, 99] (27/31)	64 [53, 75] (46/72)

Data in brackets are 95% CIs; Data in parentheses are numerators/denominators

*CRM* cystic renal mass, *AUC* area under the receiver operating characteristic curve, *PPV* positive predictive value, *NPV* negative predictive value

**Fig. 6:**
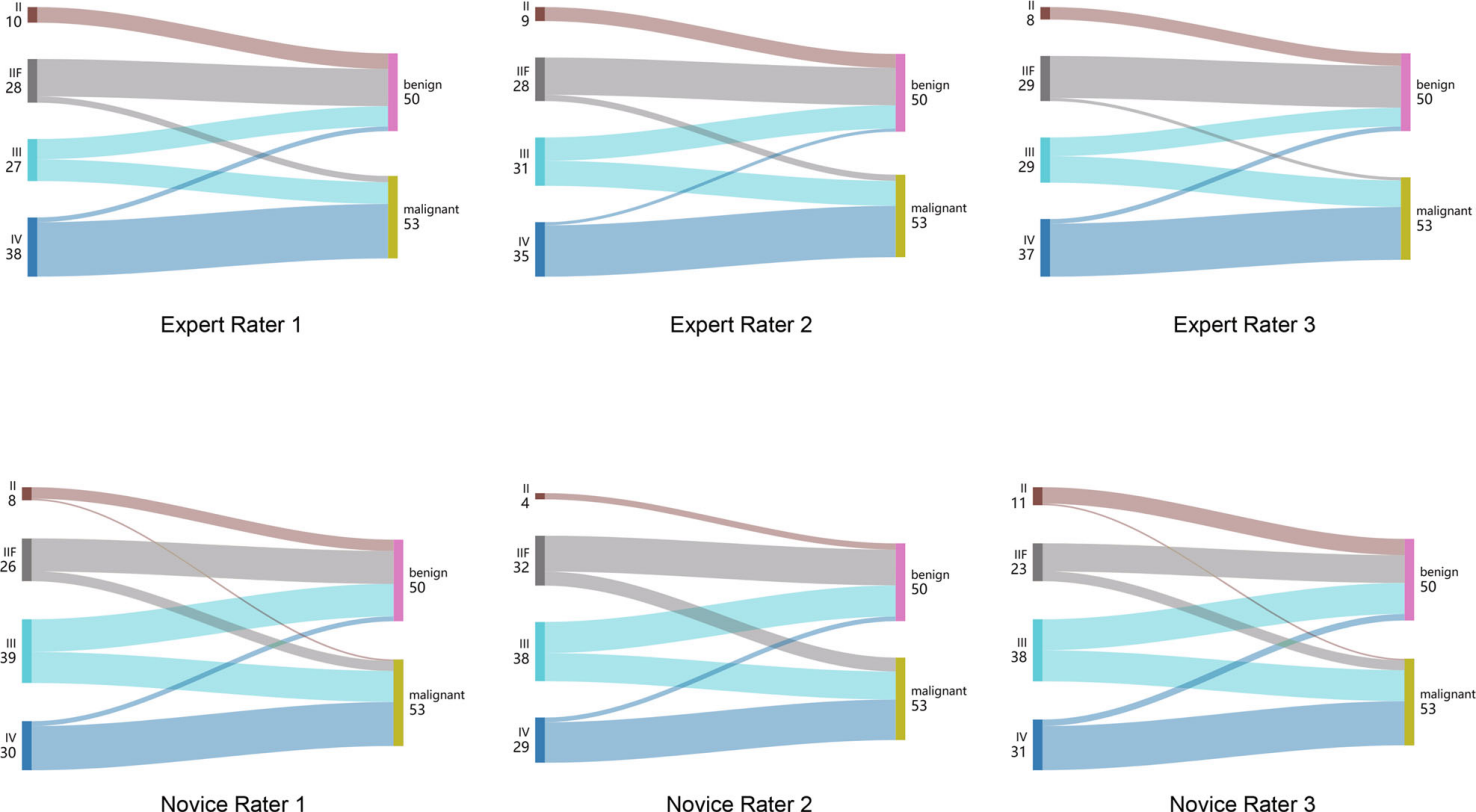
Sankey diagrams show the relationship between the Bosniak category and final diagnosis by individual raters

Sankey diagrams illustrating the association between Bosniak category assignment and final diagnosis by raters are provided in Fig. 6. There was a trend that the higher the Bosniak score of a CRM, the greater the likelihood of malignancy (*p* < 0.001 for each rater; Table S15).

Relative to BUS Bosniak classification, CEUS Bosniak classification was more likely to result in down-classification and thus greater diagnostic performance (Fig. S4 and Table S16).

## Discussion

In this study, we evaluated intra- and inter-rater agreement for detailed US features, and the Bosniak category identified confounding features and assessed diagnostic performance in predicting malignant CRMs. To date, this is the first study to investigate the reproducibility of CEUS Bosniak classification with a focus on detailed US features. For detailed US features, six raters achieved moderate to almost perfect inter-rater agreement (Gwet’s AC range, 0.44 to 0.94), with nodule (i.e., absence vs. obtuse margin vs. acute margin) being the main factor causing variability among raters. For the Bosniak category, six raters achieved substantial inter-rater agreement (Gwet’s AC = 0.75) and good diagnostic performance (AUC range, 0.78 to 0.90) in predicting malignant CRMs.

Inter-rater variability between raters in assessing the malignant potential of CRMs should be minimized regardless of raters’ experiences. High agreement between raters can guarantee the accuracy of the examination, thereby preventing over- or under-treatment [27, 28]. In our study, inter-rater agreement of the Bosniak category was substantial (Gwet’s AC = 0.75) across all raters. The inter-rater agreement for expert raters in our study (Gwet’s AC = 0.85) was similar to the published results by Münch et al [19], who reported almost perfect reliability of kappa = 0.90. In addition, both the study by Münch et al [20] and ours indicated that the inter-rater agreement for experts was superior to that for novices. The superiority of experts suggested the significant role of experience in CEUS examination, which is why EFSUMB recommends that CEUS should be conducted by radiologists with at least competence level 1 [4]. Therefore, it is essential for novices to have specialized training before performing renal CEUS examination.

With regard to the reproducibility of detailed US features, imaging feature agreement was highest for echogenic content (Gwet’s AC = 0.94), and weakest (but still of moderate agreement) for septa or wall quality (Gwet’s AC = 0.44). Similarly, a study published in 2022 on CT/MRI also emphasized component agreement [29]. It reported that component agreement was highest for calcification (Gwet’s AC = 0.94) and lowest for septa/wall thickness, number of septa, and septa /wall quality (Gwet’s AC: 0.51, 0.55, and 0.64, respectively). It is not surprising that the septa or wall quality exhibited the lowest intra- and inter-rater agreement. In the context of quantitative analysis, the difference between 3 mm and 4 mm is minimal, yet measurement deviations are unavoidable. Such deviations can significantly impact classification, with ≤ 3 mm potentially being categorized as Bosniak IIF or lower, 3–4 mm as Bosniak III, and ≥ 4 mm as Bosniak IV [4]. Furthermore, our observations found that a slight thickening frequently occurs at the junction of the cyst wall and septa. This subtle thickening is subject to varying interpretations by different raters, and we have not identified any literature providing standardized guidelines on this issue. Perhaps this represents a potential research direction for future studies. In our study, 4, 10, 9, 9, 18, and 12 individuals were assigned to Bosniak III based solely on irregularities by expert rater 1, expert rater 2, expert rater 3, novice rater 1, novice rater 2, and novice rater 3, respectively. The malignancy rates were 1/4 (25%), 1/10 (10%), 5/9 (55.6%), 3/9 (33.3%), 8/18 (44.4%), and 2/12 (16.7%), respectively, where we can saw a lot of fluctuation. This suggests that evaluating irregularities, particularly in the absence of thick septa or walls, requires extra caution. It is noteworthy that CEUS showed substantial agreement for septa number, whereas CT/MRI demonstrated only fair reliability. The difference can be explained by the higher contrast resolution of CEUS with its exceptional visualization of septal enhancement than tomographic imaging [18, 30]. What we need to be cautious about is that the high sensitivity of CEUS can lead to an upgrade compared to CT/MRI, especially in Bosniak III [12].

Future revisions to the Bosniak classification system should be guided by empirical evidence. Our study contributes such insights, revealing that disagreement in nodules was the singular multivariable predictor of variability in assigning the Bosniak category. Nodule assessment involves determining the presence or absence of a nodule and identifying whether the nodule has an obtuse or acute margin. Although CEUS Bosniak classification defines imaging features with quantitative measurements, its application can still be a challenge, and any disagreements may lead to a different Bosniak category. For example, a CRM that has a nodule ≥ 4 mm with an obtuse margin or nodule with an acute margin is classified into Bosniak IV, while a lesion that has a nodule < 4 mm with obtuse margin is classified as Bosniak III or lower [2, 4]. The assignment of the Bosniak category could be affected when there is difficulty in identifying the margin of a tiny nodule. Additional fine-tuning may be necessary to enhance inter-rater agreement without compromising predictive accuracy.

In our study, CEUS Bosniak classification showed good diagnostic performance by each rater (AUC range, 0.78–0.90). The malignancy rates for Bosniak IIF (expert 1, 14.3%; expert 2, 14.3%; expert 3, 6.9%), III (expert 1, 51.9%; expert 2, 51.6%; expert 3, 58.6%), and IV (expert 1, 92.1%; expert 2, 94.3%; expert 3, 91.9%) in the expert group are similar to those reported in the literature, whereas for the novice group, the malignancy rate for Bosniak IIF (novice 1, 22.2%; novice 2, 28.1%; novice 3, 26.1%) is higher than what has been reported [7, 24, 31]. For patients classified as Bosniak IIF by novices but malignant pathologically, the mean cyst size was smaller than that of the entire patient cohort (2.8 cm vs. 4.9 cm, *p* < 0.001). The smaller size might cause novice raters to lack confidence in the observation of details, potentially leading to a misclassification from Bosniak III/IV to Bosniak IIF.

There were several limitations. First, the study was retrospective and conducted at a single center, which limits its ability to accurately represent the real-world distribution of CRMs. Therefore, additional studies across various regions are necessary to validate the clinical value of the CEUS Bosniak classification. In addition, we didn’t compare CEUS with other imaging modalities in terms of inter-rater agreement or diagnostic effectiveness. The main focus of our study is on assessing the clinical practicality of CEUS Bosniak classification to determine whether it is suitable to complement CT for improved accuracy in malignancy assessment rather than serving as a replacement for CT.

In conclusion, CEUS Bosniak classification demonstrated moderate to almost perfect inter-rater agreement for the Bosniak category and detailed US features in the evaluation of CRMs. Disagreements in Nodule (i.e., absence vs. with obtuse margin vs. with acute margin) significantly predicted variation in Bosniak category assignment. In addition, the CEUS Bosniak classification enabled acceptable diagnostic performance. Therefore, CEUS Bosniak proves to be an accurate tool for stratifying the malignancy risk in CRMs, offering satisfactory reproducibility and diagnostic efficiency.

## Supplementary information

ELECTRONIC SUPPLEMENTARY MATERIAL

## Data Availability

The datasets used and analyzed during the current study are available from the corresponding author upon reasonable request.

## Abbreviations

AUC: Area under receiver operating characteristic curve
BUS: B-mode US
ccRCC: Clear cell renal cell carcinoma
CEUS: Contrast-enhanced US
CRM: Cystic renal mass
EFSUMB: European Federation of Societies for Ultrasound in Medicine and Biology
Gwet’s AC: Gwet’s agreement coefficient
NPV: Negative predictive value
PPV: Positive predictive value
pRCC: Papillary renal cell carcinoma
US: Ultrasound
