# Supplementary material for: Contrast-enhanced US Bosniak Classification: intra- and inter-rater agreement, confounding features, and diagnostic performance
Source: Insights Imaging. 2024 Nov 29;15:285. doi: 10.1186/s13244-024-01858-7 (PMC11607359; doi:10.1186/s13244-024-01858-7)
Supplement: Supplementary file 1 — ELECTRONIC SUPPLEMENTARY MATERIAL [file 13244_2024_1858_MOESM1_ESM.pdf]

Contrast-enhanced US Bosniak Classification: Intra- and inter-rater agreement, Confounding features, and Diagnostic performance

ELECTRONIC SUPPLEMENTARY MATERIAL

Appendix E1 Ultrasound examinations

Ultrasound examinations were conducted using either the Canon Aplio i500/900 with 8 - 1MHz convex transducer or SuperSonic Aixplorer with 6 - 1MHz convex transducer. All CRMs were scanned initially on B-mode US (BUS). For patients with more than one CRMs, we selected the most complex one as the target lesion. The side, location, size, echogenic content, calcification, septa, wall and internal nodule were described on BUS. After identifying the optimal ultrasound imaging plane, CEUS examination was conducted with a mechanical index of 0.07 after a dose of 2.4ml of SonoVue (Italy) intravenously followed by a 5ml saline flush. The target CRM was observed continuously for 1 min after administration and then intermittently scanned until 3 minutes. If needed, a second contrast agent injection was administered. Enhancement of septa/wall and nodule was assessed. All images and CEUS cine clips were stored for later analysis.

Figure S1. Contrast-enhanced US (CEUS) Bosniak classification criteria [4] (a) and its mind map for rating (b).

a

B-mode appearance		CEUS appearance	Bosniak score on multiparametric US
Simple cysts with thin wall (<2mm), sharp margins without irregularities and calcifications; anechoic content; posterior acoustic enhancement	CEUS not necessary	Thin wall without irregularities that show no enhancement on CEUS, or individual microbubbles running within tiny vessels in the wall	I
Cysts that otherwise meet the criteria of simple cysts but are characterized by 1–3 thin septa (<2mm) without irregularities. Calcifications of the wall and/or septa may be present which do not hamper evaluation of the cystic content	CEUS not necessary	Thin wall and septa without irregularities showing no enhancement, or individual microbubbles running within tiny vessels in the wall and septa	II
Cysts with internal debris, echogenic content, or mixed appearance	CEUS necessary	Thin wall and septa without irregularities showing no enhancement, or individual microbubbles running within tiny vessels in the wall and septa	II
Cysts with multiple septa, internal debris, echogenic content, or mixed appearance. Calcifications of the wall and/or septa may be present slightly hampering the evaluation of the cyst wall, content, and septa	CEUS necessary	Multiple septa, thin or minimally thickened (2–3mm). Smooth or minimally thickened wall	IIF
Totally intrarenal cysts otherwise meeting the category II criteria	CEUS necessary	Thin septa without irregularities may be present, showing no enhancement, or individual microbubbles running within tiny vessels. Differentiation between non-enhancing and enhancing wall cannot be achieved	IIF
Cysts with multiple septa, internal debris, echogenic content, or mixed appearance	CEUS necessary	Enhancing smooth thick (≥4mm) wall or septa, and/or enhancing irregular (>3mm) walls and/or septa. No nodules are seen	III
Cysts with multiple septa, internal debris, echogenic content, or mixed appearance	CEUS necessary	Enhancing smooth thick (≥4mm) wall or septa, and/or enhancing irregular (>3mm) walls and/or septa. Enhancing soft-tissue protrusions, either nodules with obtuse margins (≥4mm) or with acute margins of any size	IV

b

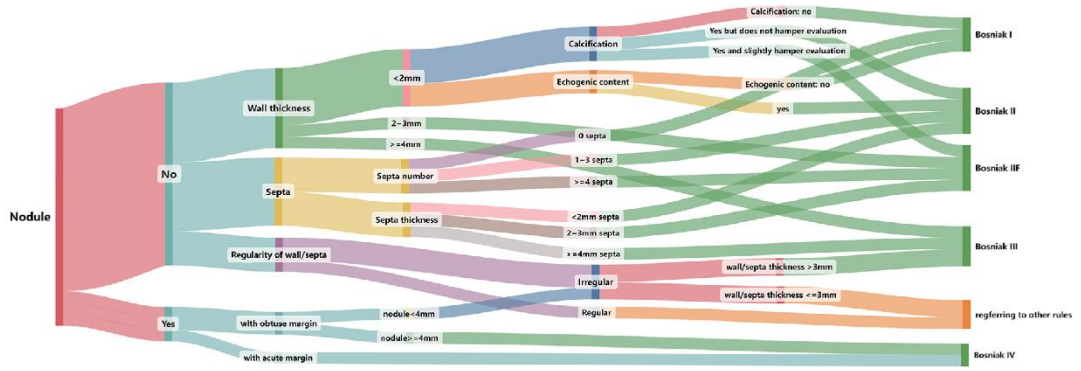

Figure S2. Linear regressions show dispersion in Bosniak category assignment as a function of dispersion in features on contrast-enhanced US. Correlations between these features and Bosniak category were very low (all P>0.05).

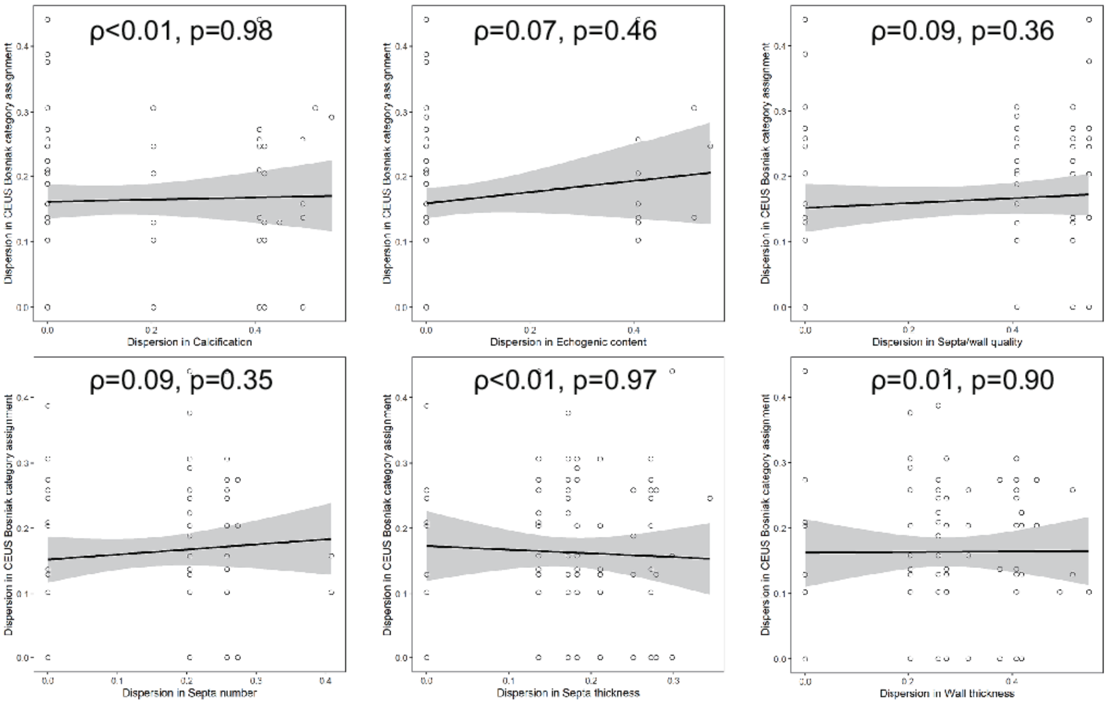

Figure S3. Radar charts show the diagnostic performance of detailed features by each rater.

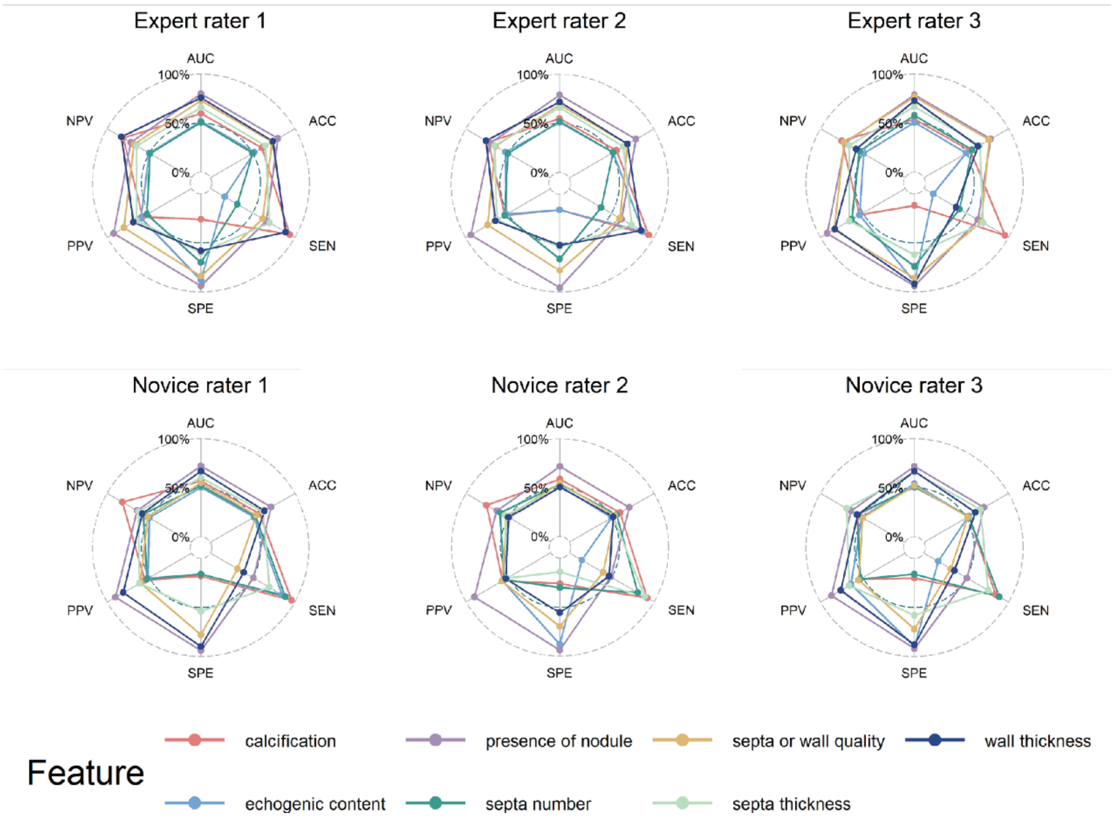

Figure S4. Sankey diagrams show change in Bosniak category by each rater between BUS Bosniak classification and CEUS Bosniak classification. BUS = B-mode US, CEUS = contrast-enhanced US.

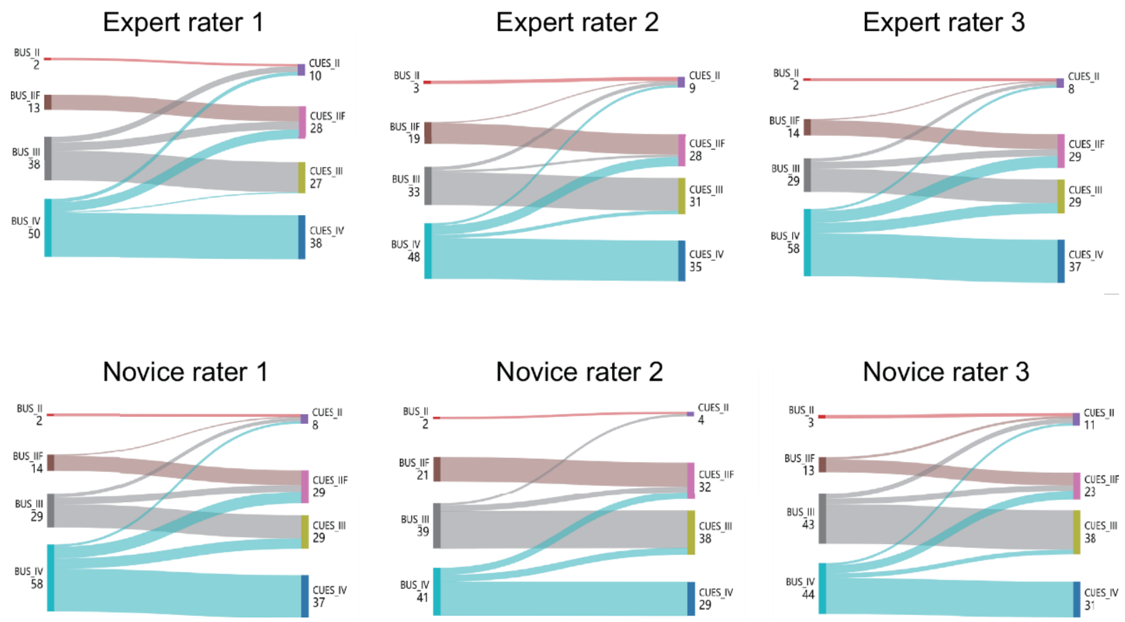

Supplemental tables

Table S1: Component Scoring and Bosniak category Assignment by raters (in the first session)						
	Expert Raters			Novice Raters		
	1	2	3	4	5	6
**BUS features**						
Septa number						
None (0)	10	11	11	9	11	9
Few (1–3) (must enhance to count)	57	50	48	54	64	49
Many (>=4) (must enhance to count)	36	42	44	40	28	45
Septa thickness						
Thin (<2mm)	36	27	31	32	25	33
Minimally thick (2-3 mm)	27	36	28	26	43	25
Thick (>=4 mm)	30	29	33	36	24	36
Wall thickness						
Thin (<2mm)	31	29	17	28	54	34
Minimally thick (2-3 mm)	48	55	60	48	33	43
Thick (>=4 mm)	24	19	26	27	16	26

Septa or wall quality						
Regular	46	51	42	71	60	59
Irregular	57	52	61	32	43	44
Nodule						
None	53	55	45	58	62	59
Nodule with acute margin	19	40	38	37	33	31
Nodule >= 4 mm with obtuse margin	31	8	20	8	8	13
Calcification						
None	87	92	95	92	86	85
Yes but doesn't hamper	6	3	2	5	5	4
Yes and slighty hamper	10	8	6	6	12	14
Echogenic content						
None	88	88	92	86	89	89
Yes	15	15	11	17	14	14
Bosniak category_BUS						
II(n)	2	3	2	4	2	3
IIF(n)	13	19	14	21	21	13

III(n)	38	33	29	33	39	43
IV(n)	50	48	58	45	41	44
**CEUS features**						
Septa number						
None (0)	16	16	18	14	13	14
Few (1–3) (must enhance to count)	55	50	50	53	65	53
Many (>=4) (must enhance to count)	32	37	35	36	25	36
Septa thickness						
Thin (<2mm)	30	25	29	29	24	28
Minimally thick (2-3 mm)	27	33	23	28	44	25
Thick (>=4 mm)	30	29	33	32	22	36
Wall thickness						
Thin (<2mm)	35	34	22	29	63	36
Minimally thick (2-3 mm)	43	49	57	46	30	42
Thick (>=4 mm)	25	20	24	28	10	25
Septa or wall quality						
Regular	62	60	60	75	67	72

Irregular	41	43	43	28	36	31
Nodule						
None	65	68	66	73	74	72
Nodule with acute margin	13	7	12	6	6	11
Nodule >= 4 mm with obtuse margin	25	28	25	24	23	20
Bosniak category_CEUS						
II(n)	10	9	8	8	4	11
IIF(n)	28	28	29	26	32	23
III(n)	27	31	29	39	38	38
IV(n)	38	35	37	30	29	31
BUS = B-mode ultrasound, CEUS = contrast enhanced ultrasound						

Table S2: Component Scoring and Bosniak category Assignment by raters (in the second session)						
	Expert Raters			Novice Raters		
	1	2	3	4	5	6
**BUS features**						
Calcification						
None	77	80	84	81	70	79
Yes but doesn't hamper	26	20	15	16	31	19
Yes and slighty hamper	0	3	4	6	2	5
Echogenic content						
None	91	89	87	90	85	86
Yes	12	14	16	13	18	17
**CEUS features**						
Septa number						
None (0)	18	15	15	19	16	13
Few (1–3) (must enhance to count)	61	52	51	46	57	46
Many (>=4) (must enhance to count)	24	36	37	38	30	44

Septa thickness						
Thin (<2mm)	32	24	30	22	29	25
Minimally thick (2-3 mm)	25	35	25	28	32	29
Thick (>=4 mm)	28	29	33	34	26	36
Wall thickness						
Thin (<2mm)	36	31	26	25	58	35
Minimally thick (2-3 mm)	45	49	63	54	31	54
Thick (>=4 mm)	22	23	14	24	14	14
Septa or wall quality						
Regular	63	57	58	69	72	67
Irregular	40	46	45	34	31	36
Nodule						
None	72	69	71	70	77	75
Nodule with acute margin	8	10	7	14	8	11
Nodule >= 4 mm with obtuse margin	23	24	25	19	18	17
Bosniak category_CEUS						
II(n)	12	8	9	5	12	6

IIF(n)	25	23	29	30	26	25
III(n)	35	38	33	35	39	44
IV(n)	31	34	32	33	26	28
BUS = B-mode ultrasound, CEUS = contrast enhanced ultrasound						

Table S3: Intrarater agreement of detailed features of each rater						
	Expert rater			Novice rater		
	1	2	3	4	5	6
Bosniak category	0.86(0.80,0.92)	0.86(0.80,0.93)	0.87(0.81,0.94)	0.82(0.76,0.89)	0.73(0.61,0.85)	0.82(0.75,0.89)
**BUS**						
Calcification	0.91(0.87,0.95)	0.91(0.85,0.96)	0.88(0.80,0.95)	0.85(0.77,0.93)	0.86(0.81,0.92)	0.81(0.71,0.91)
Echogenic content	0.91(0.84,0.98)	0.91(0.84,0.98)	0.91(0.84,0.98)	0.90(0.82,0.97)	0.87(0.78,0.95)	0.91(0.84,0.98)
**CEUS**						
Septa number	0.84(0.74,0.93)	0.83(0.73,0.93)	0.81(0.72,0.90)	0.87(0.81,0.92)	0.78(0.70,0.86)	0.81(0.74,0.88)
Septa thickness	0.90(0.80,1.00)	0.87(0.77,0.98)	0.89(0.80,0.97)	0.87(0.81,0.93)	0.85(0.79,0.92)	0.82(0.69,0.94)
Wall thickness	0.85(0.74,0.96)	0.88(0.81,0.95)	0.84(0.75,0.94)	0.83(0.75,0.90)	0.71(0.58,0.84)	0.60(0.47,0.74)
Presence of nodule	0.84(0.74,0.95)	0.84(0.74,0.95)	0.81(0.69,0.92)	0.75(0.62,0.88)	0.73(0.60,0.86)	0.79(0.67,0.90)
Nodular margin: obtuse /acute	0.88(0.80,0.96)	0.89(0.80,0.98)	0.90(0.82,0.97)	0.82(0.72,0.92)	0.84(0.74,0.93)	0.80(0.69,0.92)
Septa or wall quality	0.68(0.54,0.83)	0.64(0.49,0.79)	0.66(0.51,0.81)	0.63(0.48,0.78)	0.60(0.44,0.76)	0.64(0.48,0.79)
Note. Data are Gwet agreement coefficient; numbers in parentheses are 95% confidence intervals. BUS = B-mode ultrsdound, CEUS = contrast enhanced ultrasound.						

Table S4: Gwet agreement for all rating pairs, considering Bosniak category						
	Expert rater 1	Expert rater 2	Expert rater 3	Novice rater 1	Novice rater 2	Novice rater 3
Expert rater 1						
Expert rater 2	0.86 (0.80, 0.92)					
Expert rater 3	0.84 (0.75, 0.92)	0.87 (0.81, 0.92)				
Novice rater 1	0.73 (0.63, 0.83)	0.72 (0.62, 0.81)	0.71 (0.61, 0.81)			
Novice rater 2	0.69 (0.58, 0.80)	0.70 (0.60, 0.79)	0.71 (0.61, 0.80)	0.76 (0.68, 0.83)		
Novice rater 3	0.71 (0.60, 0.83)	0.69 (0.59, 0.79)	0.71 (0.60, 0.81)	0.80 (0.73, 0.88)	0.76 (0.69, 0.84)	
Note. Data are Gwet agreement coefficient; numbers in parentheses are 95% confidence intervals.						

Table S5: Gwet agreement for all rating pairs, considering septa number						
	Expert rater 1	Expert rater 2	Expert rater 3	Novice rater 1	Novice rater 2	Novice rater 3
Expert rater 1						
Expert rater 2	0.86 (0.79, 0.93)					
Expert rater 3	0.91 (0.86, 0.95)	0.81 (0.74, 0.89)				
Novice rater 1	0.92 (0.87, 0.96)	0.79 (0.71, 0.86)	0.86 (0.80, 0.91)			
Novice rater 2	0.86 (0.79, 0.92)	0.78 (0.69, 0.87)	0.79 (0.71, 0.86)	0.81 (0.73, 0.88)		
Novice rater 3	0.88 (0.83, 0.93)	0.85 (0.78, 0.92)	0.82 (0.76, 0.88)	0.80 (0.74, 0.86)	0.78 (0.70, 0.85)	
Note. Data are Gwet agreement coefficient; numbers in parentheses are 95% confidence intervals.						

Table S6: Gwet agreement for all rating pairs, considering septa thickness						
	Expert rater 1	Expert rater 2	Expert rater 3	Novice rater 1	Novice rater 2	Novice rater 3
Expert rater 1						
Expert rater 2	0.84 (0.79, 0.90)					
Expert rater 3	0.81 (0.74, 0.89)	0.81 (0.75, 0.88)				
Novice rater 1	0.82 (0.76, 0.88)	0.75 (0.68, 0.82)	0.77 (0.71, 0.84)			
Novice rater 2	0.72 (0.64, 0.80)	0.70 (0.61, 0.78)	0.67 (0.58, 0.76)	0.75 (0.68, 0.81)		
Novice rater 3	0.80 (0.73, 0.87)	0.76 (0.68, 0.83)	0.81 (0.74, 0.88)	0.77 (0.70, 0.84)	0.67 (0.58, 0.76)	
Note. Data are Gwet agreement coefficient; numbers in parentheses are 95% confidence intervals.						

Table S7: Gwet agreement for all rating pairs, considering wall thickness						
	Expert rater 1	Expert rater 2	Expert rater 3	Novice rater 1	Novice rater 2	Novice rater 3
Expert rater 1						
Expert rater 2	0.58 (0.44, 0.73)					
Expert rater 3	0.64 (0.51, 0.78)	0.58 (0.42, 0.74)				
Novice rater 1	0.56 (0.41, 0.72)	0.50 (0.33, 0.66)	0.56 (0.40, 0.71)			
Novice rater 2	0.43 (0.26, 0.61)	0.39 (0.20, 0.57)	0.37 (0.20, 0.55)	0.31 (0.12, 0.50)		
Novice rater 3	0.67 (0.54, 0.79)	0.54 (0.38, 0.70)	0.57 (0.39, 0.74)	0.47 (0.31, 0.62)	0.46 (0.28, 0.64)	
Note. Data are Gwet agreement coefficient; numbers in parentheses are 95% confidence intervals.						

Table S8: Gwet agreement for all rating pairs, considering presence of nodule

	Expert rater 1	Expert rater 2	Expert rater 3	Novice rater 1	Novice rater 2	Novice rater 3
Expert rater 1						
Expert rater 2	0.80 (0.69, 0.92)					
Expert rater 3	0.80 (0.69, 0.92)	0.82 (0.71, 0.93)				
Novice rater 1	0.69 (0.54, 0.83)	0.64 (0.49, 0.79)	0.64 (0.48, 0.79)			
Novice rater 2	0.60 (0.44, 0.76)	0.59 (0.43, 0.75)	0.62 (0.47, 0.78)	0.66 (0.51, 0.80)		
Novice rater 3	0.63 (0.48, 0.79)	0.59 (0.42, 0.75)	0.58 (0.42, 0.74)	0.78 (0.66, 0.90)	0.67 (0.52, 0.81)	

Note. Data are Gwet agreement coefficient; numbers in parentheses are 95% confidence intervals.

Table S9: Gwet agreement for all rating pairs, considering Nodular margin: obtuse /acute						
	Expert rater 1	Expert rater 2	Expert rater 3	Novice rater 1	Novice rater 2	Novice rater 3
Expert rater 1						
Expert rater 2	0.83 (0.73, 0.92)					
Expert rater 3	0.84 (0.75, 0.93)	0.87 (0.79, 0.95)				
Novice rater 1	0.84 (0.76, 0.93)	0.79 (0.69, 0.90)	0.77 (0.66, 0.88)			
Novice rater 2	0.77 (0.65, 0.88)	0.79 (0.69, 0.89)	0.77 (0.66, 0.88)	0.87 (0.81, 0.93)		
Novice rater 3	0.73 (0.59, 0.86)	0.73 (0.60, 0.86)	0.67 (0.52, 0.82)	0.85 (0.76, 0.94)	0.80 (0.70, 0.91)	
Note. Data are Gwet agreement coefficient; numbers in parentheses are 95% confidence intervals.						

Table S10: Gwet agreement for all rating pairs, considering septa or wall quality						
	Expert rater 1	Expert rater 2	Expert rater 3	Novice rater 1	Novice rater 2	Novice rater 3
Expert rater 1						
Expert rater 2	0.85 (0.75, 0.95)					
Expert rater 3	0.70 (0.56, 0.84)	0.66 (0.51, 0.81)				
Novice rater 1	0.35 (0.16, 0.55)	0.38 (0.19, 0.57)	0.34 (0.15, 0.54)			
Novice rater 2	0.36 (0.17, 0.55)	0.28 (0.08, 0.48)	0.28 (0.08, 0.48)	0.39 (0.20, 0.58)		
Novice rater 3	0.29 (0.09, 0.49)	0.28 (0.08, 0.48)	0.32 (0.12, 0.51)	0.85 (0.75, 0.95)	0.36 (0.16, 0.56)	
Note. Data are Gwet agreement coefficient; numbers in parentheses are 95% confidence intervals.						

Table S11: Gwet agreement for all rating pairs, considering calcification						
	Expert rater 1	Expert rater 2	Expert rater 3	Novice rater 1	Novice rater 2	Novice rater 3
Expert rater 1						
Expert rater 2	0.87 (0.79, 0.95)					
Expert rater 3	0.85 (0.77, 0.94)	0.89 (0.82, 0.96)				
Novice rater 1	0.90 (0.84, 0.97)	0.91 (0.85, 0.97)	0.93 (0.88, 0.98)			
Novice rater 2	0.85 (0.77, 0.94)	0.82 (0.72, 0.92)	0.89 (0.82, 0.96)	0.88 (0.81, 0.96)		
Novice rater 3	0.82 (0.72, 0.92)	0.85 (0.76, 0.94)	0.84 (0.75, 0.93)	0.85 (0.77, 0.94)	0.76 (0.65, 0.88)	
Note. Data are Gwet agreement coefficient; numbers in parentheses are 95% confidence intervals.						

Table S12: Gwet agreement for all rating pairs, considering echogenic content						
	Expert rater 1	Expert rater 2	Expert rater 3	Novice rater 1	Novice rater 2	Novice rater 3
Expert rater 1						
Expert rater 2	0.95 (0.90, 1.00)					
Expert rater 3	0.95 (0.90, 1.00)	0.95 (0.90, 1.00)				
Novice rater 1	0.88 (0.80, 0.96)	0.91 (0.84, 0.98)	0.94 (0.88, 0.99)			
Novice rater 2	0.96 (0.92, 1.00)	0.96 (0.92, 1.00)	0.96 (0.92, 1.00)	0.90 (0.82, 0.97)		
Novice rater 3	0.96 (0.92, 1.00)	0.94 (0.88, 0.99)	0.96 (0.92, 1.00)	0.90 (0.82, 0.97)	0.95 (0.90, 1.00)	
Note. Data are Gwet agreement coefficient; numbers in parentheses are 95% confidence intervals.						

Table S13: P values for AUC comparisons between each pair of raters						
BC_CEUS	Expert rater 1	Expert rater 2	Expert rater 3	Novice rater 1	Novice rater 2	Novice rater 3
Expert rater 1						
Expert rater 2	0.96					
Expert rater 3	0.65	0.59				
Novice rater 1	0.03	0.02	0.02			
Novice rater 2	0.01	0.01	0.01	0.56		
Novice rater 3	0.02	0.01	0.01	0.80	0.71	

Table S14: Diagnostic performance of individual features in the detection of malignancy in CRMs						
US Feature	AUC	ACC (%)	SEN (%)	SPE (%)	PPV (%)	NPV (%)
Expert rater 1						
Septa number	0.51 [0.41,0.61]	50 [50, 51] (52/103)	32 [20, 45] (17/53)	70 [57, 83] (35/50)	53 [36, 70] (17/32)	49 [38, 61] (35/71)
Septa thickness	0.66 [0.55,0.76]	65 [65, 66] (67/103)	70 [58, 82] (37/53)	60 [46, 74] (30/50)	65 [52, 77] (37/57)	65 [52, 79] (30/46)
Wall thickness	0.76 [0.67,0.84]	74 [73, 74] (76/103)	89 [80, 97] (47/53)	58 [44, 72] (29/50)	69 [58, 80] (47/68)	83 [70, 95] (29/35)
Presence of nodule	0.80 [0.73,0.87]	80 [79, 80] (82/103)	66 [53, 79] (35/53)	94 [87, 100] (47/50)	92 [84, 101] (35/38)	72 [61, 83] (47/65)
Septa or wall quality	0.73 [0.65,0.81]	73 [72, 73] (75/103)	62 [49, 75] (33/53)	84 [74, 94] (42/50)	80 [68, 93] (33/41)	68 [56, 79] (42/62)
Calcification	0.60 [0.54,0.67]	61 [61, 62] (63/103)	94 [88, 100] (50/53)	26 [14, 38] (13/50)	58 [47, 68] (50/87)	81 [62, 100] (13/16)
Echogenic content	0.52 [0.46,0.59]	52 [51, 52] (53/103)	17 [7, 27] (9/53)	88 [79, 97] (44/50)	60 [35, 85] (9/15)	50 [40, 60] (44/88)

Expert rater 2						
Septa number	0.52 [0.42,0.62]	52 [51, 52] (53/103)	38 [25, 51] (20/53)	66 [53, 79] (33/50)	54 [38, 70] (20/37)	50 [38, 62] (33/66)
Septa thickness	0.66 [0.56,0.76]	64 [64, 64] (66/103)	74 [62, 86] (39/53)	54 [40, 68] (27/50)	63 [51, 75] (39/62)	66 [51, 80] (27/41)
Wall thickness	0.72 [0.63,0.81]	69 [69, 69] (71/103)	85 [75, 94] (45/53)	52 [38, 66] (26/50)	65 [54, 76] (45/69)	76 [62, 91] (26/34)
Presence of nodule	0.79 [0.72,0.86]	79 [78, 79] (81/103)	62 [49, 75] (33/53)	96 [91, 100] (48/50)	94 [87, 102] (33/35)	71 [60, 81] (48/68)
Septa or wall quality	0.69 [0.6,0.78]	69 [69, 69] (71/103)	60 [47, 74] (32/53)	78 [66, 90] (39/50)	74 [61, 88] (32/43)	65 [53, 77] (39/60)
Calcification	0.55 [0.49,0.61]	56 [56, 57] (58/103)	94 [88, 100] (50/53)	16 [6, 26] (8/50)	54 [44, 64] (50/92)	73 [46, 99] (8/11)
Echogenic content	0.51 [0.44,0.58]	52 [52, 53] (54/103)	87 [78, 96] (46/53)	16 [6, 26] (8/50)	52 [42, 63] (46/88)	53 [28, 79] (8/15)
Expert rater 3						
Septa number	0.58 [0.48,0.68]	57 [57, 58]	42 [28, 55]	74 [62, 86]	63 [47, 79]	54 [43, 66]

		(59/103)	(22/53)	(37/50)	(22/35)	(37/68)
Septa thickness	0.67 [0.56,0.77]	66 [66, 66] (68/103)	70 [58, 82] (37/53)	62 [48, 76] (31/50)	66 [54, 78] (37/56)	66 [52, 80] (31/47)
Wall thickness	0.73 [0.64,0.81]	64 [64, 64] (66/103)	38 [25, 51] (20/53)	92 [84, 100] (46/50)	83 [68, 98] (20/24)	58 [47, 69] (46/79)
Presence of nodule	0.79 [0.72,0.86]	79 [78, 79] (81/103)	64 [51, 77] (34/53)	94 [87, 100] (47/50)	92 [83, 101] (34/37)	71 [60, 82] (47/66)
Septa or wall quality	0.77 [0.69,0.85]	77 [76, 77] (79/103)	68 [55, 80] (36/53)	86 [76, 96] (43/50)	84 [73, 95] (36/43)	72 [60, 83] (43/60)
Calcification	0.54 [0.49,0.59]	55 [55, 56] (57/103)	96 [91, 100] (51/53)	12 [3, 21] (6/50)	54 [44, 64] (51/95)	75 [45, 105] (6/8)
Echogenic content	0.51 [0.45,0.57]	50 [49, 50] (51/103)	11 [3, 20] (6/53)	90 [82, 98] (45/50)	54 [25, 84] (6/11)	49 [39, 59] (45/92)
Novice rater 1						
Septa number	0.52 [0.42,0.62]	53 [53, 54] (55/103)	89 [80, 97] (47/53)	16 [6, 26] (8/50)	53 [42, 63] (47/89)	57 [31, 83] (8/14)
Septa thickness	0.6 [0.5,0.71]	62 [62, 63]	70 [58, 82]	54 [40, 68]	62 [49, 74]	63 [48, 77]

		(64/103)	(37/53)	(27/50)	(37/60)	(27/43)
Wall thickness	0.67 [0.58,0.77]	64 [64, 64] (66/103)	40 [26, 53] (21/53)	90 [82, 98] (45/50)	81 [66, 96] (21/26)	58 [47, 69] (45/77)
Presence of nodule	0.72 [0.65,0.8]	72 [72, 72] (74/103)	51 [38, 64] (27/53)	94 [87, 100] (47/50)	90 [79, 101] (27/30)	64 [53, 75] (47/73)
Septa or wall quality	0.55 [0.46,0.64]	54 [54, 55] (56/103)	32 [20, 45] (17/53)	78 [66, 90] (39/50)	61 [43, 79] (17/28)	52 [41, 63] (39/75)
Calcification	0.57 [0.51,0.63]	58 [58, 59] (60/103)	96 [91, 100] (51/53)	18 [7, 29] (9/50)	55 [45, 66] (51/92)	82 [59, 105] (9/11)
Echogenic content	0.5 [0.43,0.58]	52 [51, 52] (53/103)	85 [75, 94] (45/53)	16 [6, 26] (8/50)	52 [41, 62] (45/87)	50 [26, 74] (8/16)
Novice rater 2						
Septa number	0.54 [0.44,0.64]	56 [56, 57] (58/103)	81 [71, 92] (43/53)	30 [17, 43] (15/50)	55 [44, 66] (43/78)	60 [41, 79] (15/25)
Septa thickness	0.51 [0.41,0.62]	52 [52, 53] (54/103)	89 [80, 97] (47/53)	14 [4, 24] (7/50)	52 [42, 62] (47/90)	54 [27, 81] (7/13)
Wall thickness	0.51 [0.41,0.61]	52 [51, 52]	47 [34, 61]	56 [42, 70]	53 [39, 68]	50 [37, 63]

		(53/103)	(25/53)	(28/50)	(25/47)	(28/56)
Presence of nodule	0.72 [0.64,0.79]	71 [70, 71] (73/103)	49 [36, 62] (26/53)	94 [87, 100] (47/50)	90 [79, 101] (26/29)	64 [52, 74] (47/74)
Septa or wall quality	0.55 [0.46,0.64]	54 [54, 55] (56/103)	40 [26, 53] (21/53)	70 [57, 83] (35/50)	58 [42, 74] (21/36)	52 [40, 64] (35/67)
Calcification	0.59 [0.52,0.66]	60 [60, 61] (62/103)	92 [85, 100] (49/53)	26 [14, 38] (13/50)	57 [47, 67] (49/86)	76 [56, 97] (13/17)
Echogenic content	0.52 [0.45,0.58]	50 [50, 51] (52/103)	15 [6, 25] (8/53)	88 [79, 97] (44/50)	57 [31, 83] (8/14)	49 [39, 60] (44/89)
Novice rater 3						
Septa number	0.51 [0.4,0.61]	53 [53, 54] (55/103)	89 [80, 97] (47/53)	16 [6, 26] (8/50)	53 [42, 63] (47/89)	57 [31, 83] (8/14)
Septa thickness	0.66 [0.56,0.76]	67 [67, 67] (69/103)	76 [64, 87] (40/53)	58 [44, 72] (29/50)	66 [54, 78] (40/61)	69 [55, 83] (29/42)
Wall thickness	0.67 [0.57,0.76]	61 [61, 62] (63/103)	36 [23, 49] (19/53)	88 [79, 97] (44/50)	76 [59, 93] (19/25)	56 [45, 67] (44/78)
Presence of nodule	0.72 [0.64,0.79]	71 [70, 71]	51 [38, 64]	92 [84, 100]	87 [75, 99]	64 [53, 75]

		(73/103)	(27/53)	(46/50)	(27/31)	(46/72)
Septa or wall quality	0.52 [0.43,0.61]	52 [51, 52] (53/103)	32 [20, 45] (17/53)	72 [60, 84] (36/50)	55 [37, 72] (17/31)	50 [38, 62] (36/72)
Calcification	0.52 [0.45,0.6]	53 [53, 54] (55/103)	85 [75, 94] (45/53)	20 [9, 31] (10/50)	53 [42, 64] (45/85)	56 [33, 78] (10/18)
Echogenic content	0.54 [0.47,0.6]	52 [52, 53] (54/103)	17 [7, 27] (9/53)	90 [82, 98] (45/50)	64 [39, 89] (9/14)	51 [40, 61] (45/89)

Note. Data in brackets are 95% confidence intervals; Data in parentheses are numerators/denominators. CRM = cystic renal mass, AUC = area under the receiver operating characteristic curve, ACC = accuracy, SEN = sensitivity, SPE = specificity, PPV = positive predictive value, NPV = negative predictive value.

Table S15: Malignancy rate of different Bosniak category by each rater						
Rater	Malignancy rate (%)				X^2	*P*
	**Bosniak II**	**Bosniak IIF**	**Bosniak III**	**Bosniak IV**		
Expert rater						
Expert rater 1	0.0	14.3	51.9	92.1	51.2	<0.001
Expert rater 2	0.0	14.3	51.6	94.3	50.7	<0.001
Expert rater 3	0.0	6.9	58.6	91.9	56.3	<0.001
Novice rater						
Novice rater 1	12.5	22.2	50.0	90.0	32.0	<0.001
Novice rater 2	0.0	28.1	47.4	89.7	28.4	<0.001
Novice rater 3	9.1	26.1	50.0	87.1	29.6	<0.001

Table S16: Comparison of the AUC of BUS and CEUS criterion among each rater			
	**AUC_BUS**	**AUC_CEUS**	**P value**
Expert rater 1	0.71	0.89	<0.001
Expert rater 2	0.72	0.89	<0.001
Expert rater 3	0.74	0.90	<0.001
Novice rater 1	0.67	0.80	<0.001
Novice rater 2	0.62	0.78	<0.001
Novice rater 3	0.62	0.79	<0.001
